# From Traditional Resource to Global Commodities:—A Comparison of *Rhodiola* Species Using NMR Spectroscopy—Metabolomics and HPTLC

**DOI:** 10.3389/fphar.2016.00254

**Published:** 2016-08-29

**Authors:** Anthony Booker, Lixiang Zhai, Christina Gkouva, Shuyuan Li, Michael Heinrich

**Affiliations:** ^1^Research Cluster Biodiversity and Medicines/Centre for Pharmacognosy and Phytotherapy, UCL School of Pharmacy, University of LondonLondon, UK; ^2^Division of Herbal and East Asian Medicine, Department of Life Sciences, University of WestminsterLondon, UK; ^3^Department of Traditional Chinese Medicine, School of Traditional Chinese Medicine, Guangdong Pharmaceutical UniversityGuangzhou, China

**Keywords:** *Rhodiola*, metabolomics, herb quality, adulteration, HPTLC, NMR

## Abstract

The fast developing international trade of products based on traditional knowledge and their value chains has become an important aspect of the ethnopharmacological debate. The structure and diversity of value chains and their impact on the phytochemical composition of herbal medicinal products, as well as the underlying government policies and regulations, have been overlooked in the debate about quality problems in transnational trade. *Rhodiola* species, including *Rhodiola rosea* L. and *Rhodiola crenulata* (Hook. f. & Thomson) H. Ohba, are used as traditional herbal medicines. Faced with resource depletion and environment destruction, *R. rosea* and *R. crenulata* are becoming endangered, making them more economically valuable to collectors and middlemen, and also increasing the risk of adulteration and low quality. *Rhodiola* products have been subject to adulteration and we recently assessed 39 commercial products for their composition and quality. However, the range of *Rhodiola* species potentially implicated has not been assessed. Also, the ability of selected analytical techniques in differentiating these species is not known yet. Using a strategy previously developed by our group, we compare the phytochemical differences among *Rhodiola* raw materials available on the market to provide a practical method for the identification of different *Rhodiola species* from Europe and Asia and the detection of potential adulterants. Nuclear magnetic resonance spectroscopy coupled with multivariate analysis software and high performance thin layer chromatography techniques were used to analyse the samples. Rosavin and rosarin were mainly present in *R. rosea* but also in *Rosea sachalinensis* Borris. 30% of the *Rhodiola* samples purchased from the Chinese market were adulterated by other *Rhodiola* spp. The utilization of a combined platform based on ^1^H-NMR and HPTLC methods resulted in an integrated analysis of different *Rhodiola* species. We identified adulteration at the earliest stage of the value chains, i.e., during collection as a key problem involving several species. This project also highlights the need to further study the links between producers and consumers in national and trans-national trade.

## Introduction

While medicinal plants and spices have been traded for centuries on a global scale, the fast developing international trade of products now includes a large number of species which are used based on local and traditional knowledge and practice. The value chains of such products are starting to become an important topic in the ethnopharmacological debate. The structure and diversity of value chains, as well as their impact on the phytochemical composition of herbal medicinal products (HMPs) has been overlooked in quality issues in transnational trade. Different government policies and regulations governing trade in herbal medicinal products impact on such value chains.

**Figure 1:**
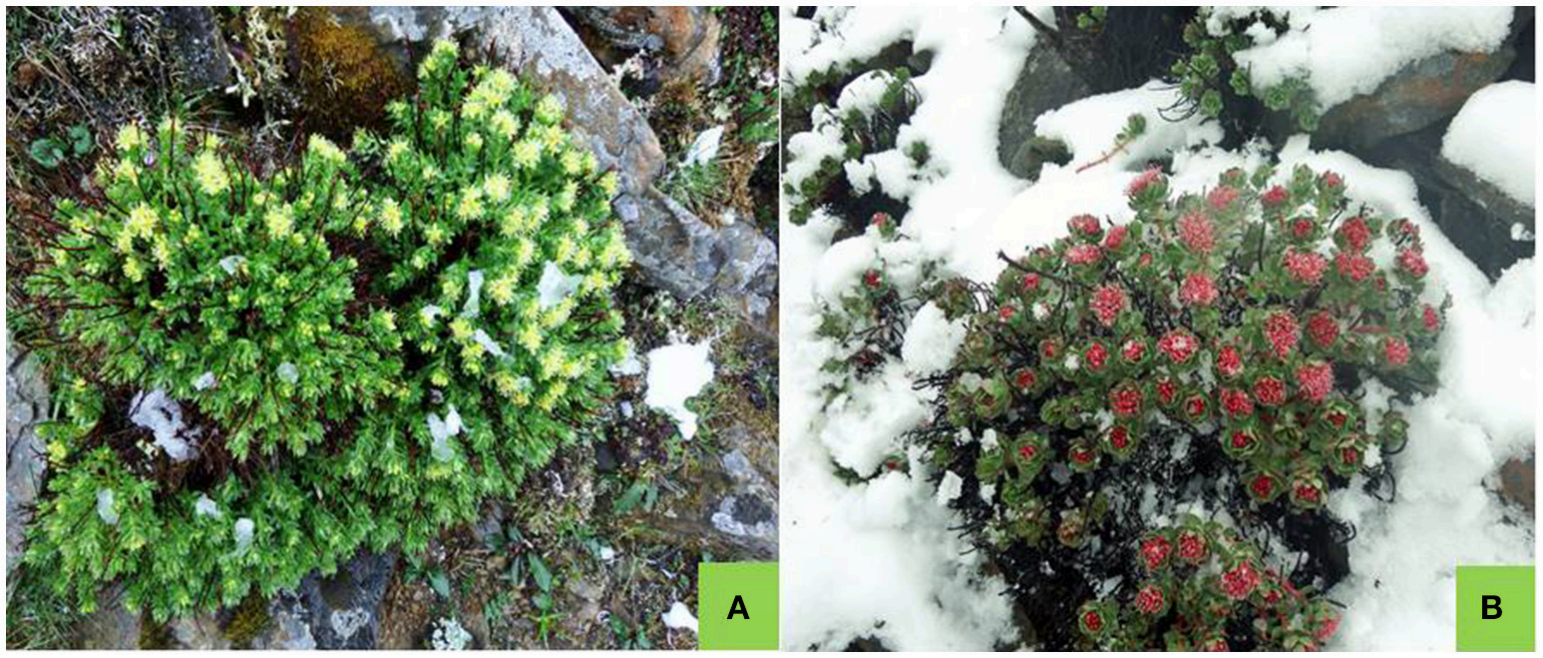
***Rhodiola* species**. **(A)** *R. rosea*; **(B)** *R. crenulata*. Photos taken by A. Booker, Sichuan-Tibet border, June, 2015.

Medicinal *Rhodiola* species, including *Rhodiola rosea* L. and *Rhodiola crenulata* (Hook. f. & Thomson) H. Ohba (Figure 1), have been used widely in Europe and Asia as traditional herbal medicines with numerous claims for their therapeutic effects. Faced with resource depletion and environment destruction, *R. rosea* and *R. crenulata* are becoming endangered, making them more economically valuable to collectors and middlemen, and also increasing the risk of adulteration and low quality. Poor quality and adulterated *R. rosea* products have been previously reported (Booker et al., 2015; Xin et al., 2015) and this paper investigates some aspects of the value chains that leads to the production of such products.

Adulteration of *R. rosea* products with *R. crenulata* has been previously reported but our fieldwork investigations suggested that other species may be implicated, and particularly *Rhodiola sachalinensis*, another species that appears to contain rosavins (the main marker compounds used for the identification of *R. rosea*).

The genus *Rhodiola* (Crassulaceae) comprises ~90 species of succulent and herbaceous perennial plants, which mainly show a circumpolar distribution across the northern hemisphere (Xia et al., 2005; Lu and Lan, 2013). *Rhodiola* species usually grow in mountainous areas such as rock ledges, precipices, tundra, brooks, and river banks (Zhu and Lou, 2010).

### Ethnopharmacological importance of key *Rhodiola* species

In Europe and North America, *Sedum roseum (L.)* Scop. (commonly named under its synonym *R. rosea* L.) is the most well-known and widely used among the different species. It is also known as golden root, or artic root which reputedly demonstrates the economic importance and the geographical distribution of the plant. It has a rich history of traditional use in Russia, Europe and Asia with various uses according to the region (e.g., as shown in Table 1).

**Table 1:** **Traditional uses of *R. rosea* in different regions**.

**Region**	**Use**	**References**
Russia	Escalation of physical endurance Remedy against fatigue and high altitude sickness Aphrodisiac	Shikov et al., 2014; Alm, 2004
Norway	Astringent Cure for scurvy Remedy against hair-loss and urinary tract disorders	Alm, 2004
Iceland and Denmark	Alleviation of headaches	Alm, 2004
France	Stimulant Astringent	Panossian et al., 2010
Alaska	Cure for sores Remedy against tuberculosis	Alm, 2004
Mongolia	Remedy against tuberculosis Anticancer Escalation of physical endurance Treatment for lung inflammation	Brown et al., 2002; World Health Organization, 2013

In Europe, the first documented medicinal use of *R. rosea* can be traced back to Dioscorides in 77 A.D. (Brown et al., 2002). In C. v. Linne's *Materia Medica*, the root of *R. rosea* was recommended for several conditions such as headaches, “hysteria,” hernias and discharges (C. v. Linne, 1749 in Panossian et al., 2010). Throughout the years, it has appeared in many pharmacopeias and medicinal books of different countries such as Sweden, France, Norway, Germany, Iceland, Estonia, and Russia (Brown et al., 2002; Alm, 2004; Panossian et al., 2010; Shikov et al., 2014).

In China, 73 different *Rhodiola* species have been reported, mainly in the northwest and southwest regions such as Tibet and the Sichuan province. The adaptogenic and tonic properties of the *Rhodiola* plants have been widely used in traditional Chinese and Tibetan medicine (Li and Zhang, 2008). They are generally referred to with the Pinyin name Hong Jing Tian 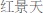 [red (or glorious) view of heaven] with slight alterations for each species (Table 2).

**Table 2:** **Examples of the similar Pin Yin names of different *Rhodiola* species in China**.

**Scientific name**	**Pin Yin name**
*R. rosea* L.	Qiang Wei (rose smell) Hong Jing Tian
*R. sachalinensis* Borris.	Gao Shan (high mountain) Hong Jing Tian
*R. quadrifida* (Pall.) Fisch. & C.A.Mey	Si Lie (four split) Hong Jing Tian
*R. crenulata* (Hook. f. & Thomson) H. Ohba	Da Hua (big flower) Hong Jing Tian
*R. yunnanensis* (Franch.) S.H. Fu	Yunnan (From Yunnan) Hong Jing Tian
*R. kirilowii* (Regel) Maxim.	Xia Ye (narrow leaf) Hong Jing Tian
*R. fastigiata* (Hook. f. & Thomson) S.H. Fu	Chang Bian (clustered) Hong Jing Tian

*R. crenulata* can be traced back to Tibetan medicine books including “The Four Medical Tantras” (*rgyud-bzhi* in Tibetan, *Si Bu Yi Dian* in Chinese), Yue Wang's Classical Medicinal Book (*Somaratsa* in Tibetan, *Yue Wang Yao Zhen* in Chinese), and Jing Zhu Materia Medica [*Shel Gong Shel Phreng* in Tibetan*, Jing Zhu Ben Cao* in Chinese (Lu and Lan, 2013)]. It is used for treatment of cough, hemoptysis, pneumonia, and abnormal vaginal discharge. In Traditional Chinese Medicine (TCM), it has effects of nourishing qi as well as promoting blood circulation and is mainly prescribed for qi deficiency and blood stasis (QDBS), stroke, hemiplegia, and fatigue. It is commonly used in China and Tibet for treating altitude sickness.

### Phytochemical and pharmacological research

Research on the phytochemistry and pharmacology of *Rhodiola* spp. was initiated in the 1960s in the Soviet Union and Scandinavia, mainly focusing on *R. rosea* (Brown et al., 2002). After the turn of the century the interest in this plant spread globally. Intensive phytochemical research led to the detection of known and novel compounds in *R. rosea* and related species (Ma et al., 2006; Yousef et al., 2006). Between 2000 and 2015 an increased number of publications stemming from Asian research groups have focused on the detection of novel compounds from *Rhodiola* species, usually in combination with their respective pharmacological assessments (Fan et al., 2001; Nakamura et al., 2007, 2008).

There are more than a few hundred pharmacological studies on medicinal *Rhodiola species* (mainly on *R. rosea)* that show a wide range of activities reflecting their diverse traditional use. They possess adaptogenic and stress-protective (neuro-cardio and hepato protective) and antioxidant effects, as well as stimulating effects on the central nervous system, including on cognitive functions such as attention, memory and learning; anti-fatigue effects; antidepressive and anxiolytic effects; endocrine activity normalizing; and life-span increasing effects (Aslanyan et al., 2010; Sarris et al., 2011; Panossian et al., 2013). The main active compounds are reputedly phenylpropanoids (rosavin, rosarin, rosin) and phenylethanoids (salidroside and tyrosol).

### Quality issues of medicinal *Rhodiola* spp.

*Rhodiola* roots and rhizomes are highly valuable products traded at an international level. Since the majority of *R. rosea* and *R. crenulata* raw material supplied still comes from wild-collection, their intensive collection leads to scarcity (Galambosi, 2006; Lu and Lan, 2013).

Herbal preparations of *Rhodiola* species (mainly *R. rosea*) are extensively utilized around the globe. There is an increasing number of commercial products available on the American, Asian and European markets, either as food supplements or herbal medicines. *R. rosea* herbal monographs have been included in many Pharmacopeias worldwide. On the other hand, *R. crenulata* is the only species used medicinally in TCM (Table 3).

**Table 3:** **Generation of *Rhodiola* spp. recorded in selected pharmacopeias and publications**.

**Pharmacopeia/publication**	**Recorded *Rhodiola* species**	**Medicinal use part**	**Herbal product**
Department of Health and Ageing, Australian Government	*Rhodiola rosea*	Root (Rhizome)	Dry extract
Committee on Herbal Medicinal Products, 2012	*Rhodiola rosea*	Rhizoma et radix	Extract
United States Pharmacopeia (32th Edition)	*Rhodiola rosea*	Rhizoma et radix	Dry extract, tincture
Chinese Pharmacopoeia, 2010	*Rhodiola crenulata*	Rhizoma et radix	Extract
Russian Pharmacopoeia (12th Edition)	*Rhodiola rosea*	Rhizoma et radix	Extract

Due to this rapid increase of *Rhodiola* raw material demand, other *Rhodiola* species such as *R. fastigiata, R. sachalinensis, R. quadrifida, Rhodiola sacra* (Prain ex Hamet) S. H. Fu and *Rhodiola serrata* H. Ohba have been sold on the market (Xin et al., 2015). Since there is not any consistent worldwide quality control programme, inadequate quality assessment of *Rhodiola* spp. is a common issue. This raises concerns about possible adulteration and misidentification issues. The lack of genuine drug material, confusion over the Chinese Pin Yin name of the drug when sourcing from China and accidental or deliberate adulteration during the manufacturing stage may contribute to low quality of final products.

The analytical techniques currently available focus on identifying *R. rosea* or *R. crenulata* through chromatographic methods. Other species of *Rhodiola* have generally not been considered. *R. sachalinensis* presents a particular problem as it may contain similar marker compounds to *R. rosea* (and some sources suggest that it is the same species—see http://www.kew.org/mpns-portal).

### Integrated analytical platform approach

#### NMR-based metabolomics

NMR-based metabolic fingerprinting has been used in the analysis of numerous food and medicinal species focusing on their quality assurance as well as their pharmacology. Such comparative studies include Danggui [*Angelica sinensis* (Oliv.) Diels] and Engelwurz/European Angelica (*Angelica archangelica* L.; Li et al., 2014). Metabolomic differences between different *Tussilago farfara* L. accessions (Zhi et al., 2012) and different *Salvia miltiorrhiza* Bunge production sites (Jiang et al., 2014) were also studied by NMR fingerprinting coupled with multivariate analysis. Compared to GC-MS and LC-MS, NMR has some advantages such as non-selectiveness, high reproducibility, and good stability (Simmler et al., 2014). At the same time, structural information on metabolites can be obtained from NMR directly. Therefore, NMR can be regarded as an ideal choice for chemical comparison and identification of the phytochemical differences of medicinal plants.

#### HPTLC

Since the NMR-metabolomic approach is not a validated pharmacopoeial method, there is a need to be compared to a standard method like high performance thin layer chromatography (HPTLC). This method is widely used for the authentication and quality control of herbal substances (Reich et al., 2008). Compared to NMR-based metabolic fingerprinting, HPTLC could be highly effective with relatively lower price (Booker et al., 2014). HPTLC can also be helpful for the identification of specific compounds. Therefore, we chose these two complementary approaches in this study.

A third analysis strategy using DNA bar coding was used to help verify some of the samples (details are given in the Supplement S2).

## Materials and methods

### Sampling and preparation of plant material

Forty-two batches of *Rhodiola* market samples (i.e., not authenticated) were collected between October 2014 and January 2015 from different suppliers including retail outlets, The internet, pharmaceutical companies in seven different locations (Beijing, Guangdong, Qinghai, Anhui, Hebei, Jilin, and Hong Kong SAR) and in China, Germany and Russia. These raw-material samples were mainly labeled as *R. rosea, R. crenulata, R. sachalinensis*, and *R. quadrifida*. 18 batches of authenticated plant material were provided by Agroscope Institute (Switzerland). The samples were rhizomes of *R. rosea* plants propagated from different wild Swiss populations (Mattmark, Carrasino, and Nomnon) or botanical gardens (Switzerland and Germany). In addition, authenticated *R. rosea* samples which were grown from seeds or provided to the institute by Dr. Bertalan Galambosi were also included. Lastly, in June 2015, samples of *R. crenulata* and *R. fastigiata* roots and rhizomes were collected from Garze, Sichuan, China (altitude 4500 m). These samples were authenticated by Professor Shuyuan Li, (Guangdong Pharmaceutical University, Guangzhou, China). Botanical reference materials (BRMs) for *R. rosea, R. crenulata*, and *R. sachalinensis* were obtained from the National Institute of Food and Drug Control (NIFDC, China), Dr. William Schwabe (Germany) and Agroscope (Switzerland). BRMs for *R. quadrifida* and *R. fastigiata* were provided by Professor Alexander Shikov (Saint-Petersburg Institute of Pharmacy, Russia) and Dr. Anthony Booker (UCL School of Pharmacy). *R. fastigiata* was authenticated by Professor Shuyuan Li (Guangdong Pharmaceutical University, Guangzhou, China).

All the collected samples were deposited in the herbarium of the UCL School of Pharmacy (London, UK). A detailed description of the investigated samples including their origins and representative symbols are provided in Supplement (S1).

Crude root samples were ground to powder using a household grinder (EK1665ROFOB, Salter, UK) and sieved (0.70 mm mesh). All the powder samples were kept in 1.5 ml tubes (Eppendorf AG.) at 4°C until use.

### Solvents, reagents, and reference compounds

Deuterium oxide (D_2_O), methanol-d_4_ (99.8% D, MeOD), dimethyl sulfoxide-d_6_ (DMSO-d_6_), and tetramethylsilane (TMS) were obtained from Cambridge Isotope Laboratories Inc. (Andover, MA). Salidroside, gallic acid, rosarin, and rosavin were purchased from Sigma-Aldrich Chemicals (St Luis, USA). Tyrosol was purchased from Acros organics (New Jersey, US). Water used in this study was purified by using ULTRAPURE water system (Millipore, Germany). All other chemicals were of analytical grade.

### ^1^H-NMR spectroscopy

#### Sample preparation

Nine-hundred microliter of MeOD-d_4_ was added for extraction. The samples were vortexed (Rodamixer, UK) for 30 s and sonicated at an ultrasound bath (Fisher, XB22, UK) for 10 min. The solutions were centrifuged for 10 min at 14,000 rpm (EBA21, Hettich, Faust Laborbedarf AG, Germany). Six-hundred microliter of supernatant was transferred to a 5 mm diameter NMR spectroscopy tube and the samples were submitted for NMR spectroscopic analysis. The one and two dimensional ^1^H-NMR spectra were recorded on Brucker Avance 500 MHz spectrometer (Bruker Analytic, Germany), which was equipped with a QNP (^31^P, ^13^C, ^15^N, and ^1^H) 5 mm cryoprobe. The acquisition parameters were: size of the spectra 64 k data points, line broadening factor = 0.16 Hz, pulse width (PW) = 30 degrees, and the relaxation delay d1 = 1 s. The acquisition temperature was 298 K.

In order to assess the coherence of the results obtained, two samples from the same batch were subjected to NMR analysis on the different days of examination. To minimize the error caused by root selection during sample grinding, any samples weighing more than 500 g were analyzed twice.

#### Data analysis

The resulting spectra were manually phased and auto-baseline corrected by Topspin 3.2 (Bruker, Germany) for organic fractions. Signals between δ 5.20–4.40 ppm and δ 3.35–3.22 ppm were removed prior to statistical analysis due to the presence of methanol-d_4_. The total area of peaks (δ 10.00–0.00 ppm) was integrated into small (0.04 ppm) buckets by bucketing (binning) function using AMIX or ACD-Labs in order to generate a number of integrated regions of the data set. The buckets obtained were then imported to Microsoft EXCEL (2013) where the samples were re-labeled and their species information was added.

Principal component analysis (PCA) was performed with SIMCA-P 13.0 (Umetrics, Umeå, Sweden) for metabolomic analysis of the generated dataset. Scaling mode of Pareto (Par) and Unit Variance (UV) were tested to optimize the analysis model.

### HPTLC

#### Sample preparation

One milliliter of ethanol was added to 50 mg of weighed samples for extraction. The solutions were then mixed on a rotary mixer (Rodamixer, UK) for 30 s, sonicated in an ultrasound bath (Fisher, XB22, UK) for 10 min and centrifuged for 10 min at 14,000 rpm. The supernatant was used for HPTLC analysis. The reference standard solutions of salidroside, rosarin, rosavin, gallic acid, and tyrosol were prepared at a concentration 1 mg/ml in methanol. Both the reference material and the test samples were stored at 4°C.

#### Data analysis

Samples were applied to the plates as bands 8 mm wide by using Linomat 5 semi-automatic applicator with 100 μl syringe. The space between bands was 2.0 mm and the rate of application was 90 nl·s^−1.^ The number of tracks per plate was 15, and 5 μl of standard and sample solutions were applied.

The temperature and relative humidity were controlled to 21–24°C and 33%, respectively. Ten milliliter of solvent was poured into the right inlet for development and 25 ml of solvent was poured into the left inlet for saturation. Plates were previously air dried for 10 s and developed in a 20 × 10 cm twin-trough chamber (Analtech, USA) lined with Whatman filter paper (20 × 10 cm) and saturated with mobile phase (Ethylacetate, methanol, water, formic acid (77:13:10:2) vapor for 20 min. The development distance was 70.0 mm from the lower edge.

The developed plates were derivatised by dipping in sulfuric acid reagent, using a CAMAG chromatogram immersion device and heated at 100°C on a plate heater for 5 min. Sulfuric acid reagent was prepared with a procedure as follow: 20 ml sulfuric acid was carefully added to 180 ml ice-cold methanol and mixed. The plates were visualized using CAMAG visualizer under white light, UV 254 nm and at UV 366 nm, photographed and uploaded to HPTLC computer software (VisionCats).

## Results and discussion

### ^1^H-NMR and multivariate statistical analysis

**Figure 2:**
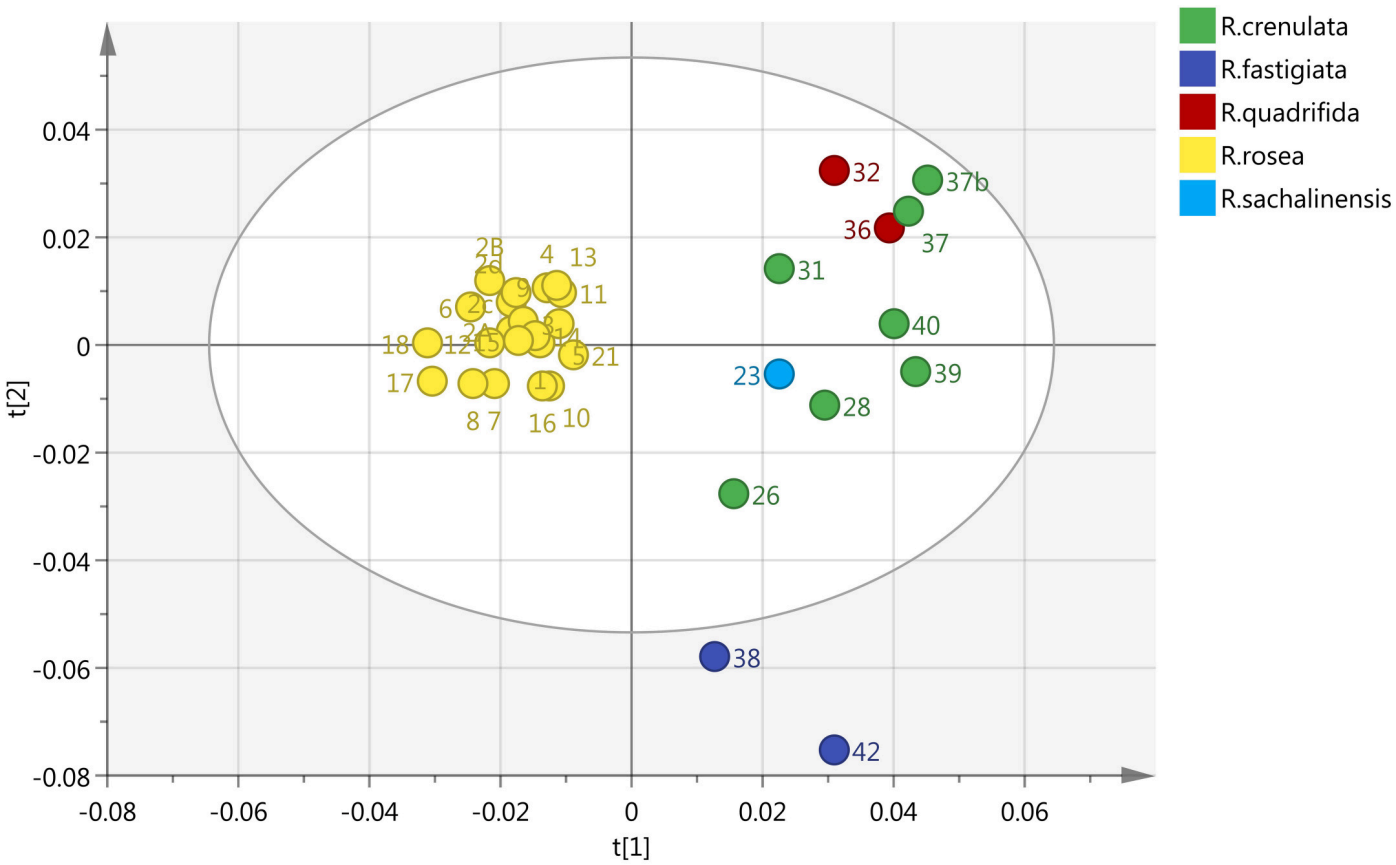
**Scores plot of five different species of *Rhodiola* (*R. rosea*, *R. crenulata*, *R. quadrifida*, *R. sachalinensis*, *R. fastigiata*), showing principle component 1 and principal component 2**.

By incorporating the whole region (0–10 ppm) and Pareto (Par) scaling, a significant clustering is observed in *R. rosea* samples (Figure 2). *R. rosea* can be differentiated distinctly from the rest of the species based on their principal component variability.

**Figure 3:**
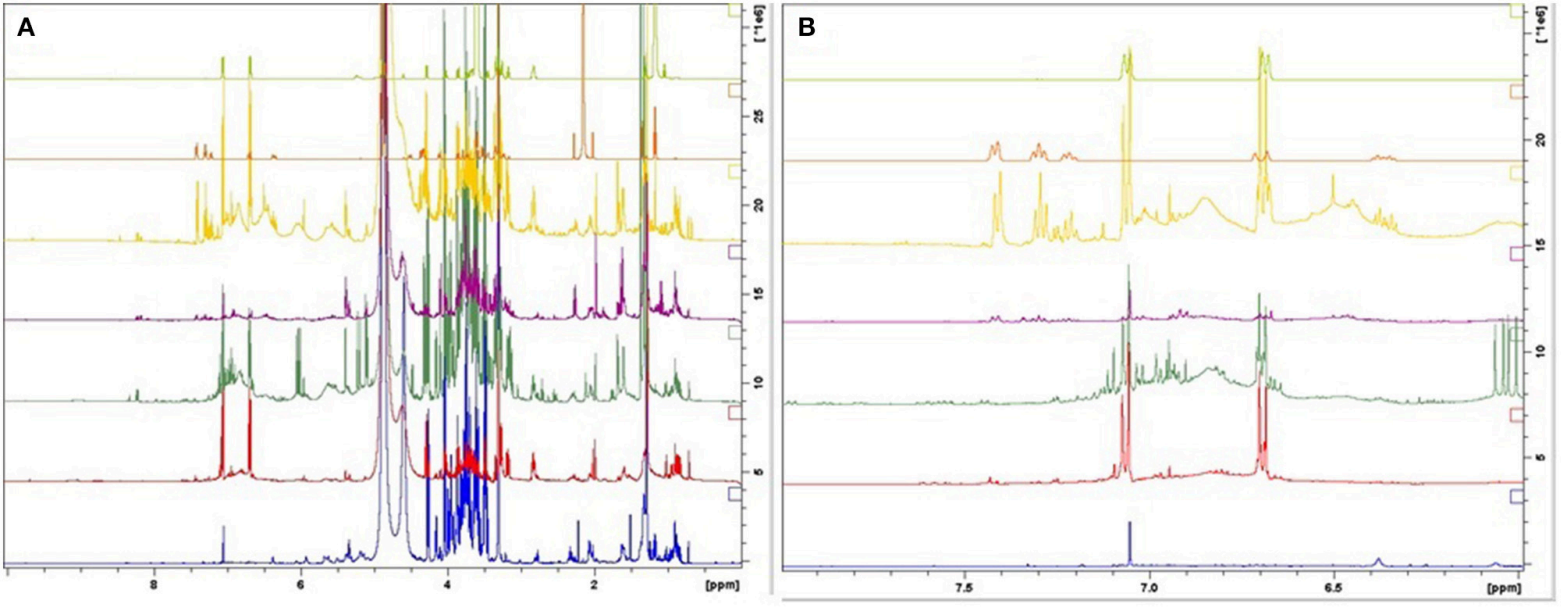
**^1^H-NMR spectra of the reference compounds, salidroside and rosavin, together with the spectra of botanical reference material**. 1: *R. fastigiata*, 2: *R. quadrifida*, 3: *R. crenulata*, 4: *R. sachalinensis*, 5: *R. rosea*, 6: rosavin, and 7: salidroside. (From bottom to top) **(A)** Whole region (0–10 ppm); **(B)** aromatic region (6–8 ppm).

**Figure 4:**
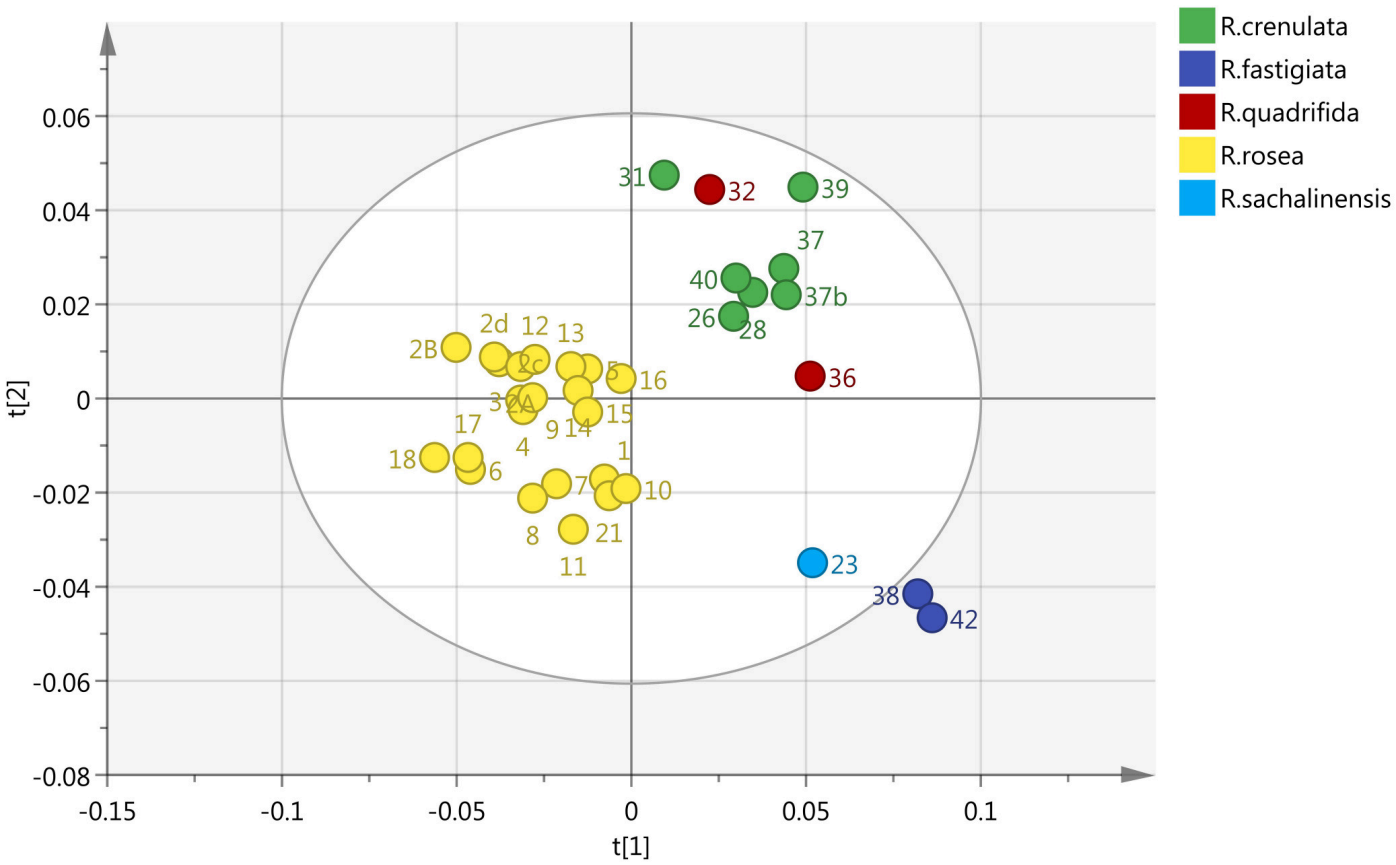
**Scores plot of *Rhodiola* samples using the aromatic ^1^H-NMR region and Pareto scaling**.

According to the spectra of the species (Figure 3), the aromatic region (6–8 ppm) is dominated by the main marker compounds (rosavin and salidroside). Hence, this region was analyzed independently using Par scaling (Figure 4). Based on the scores plot produced, *Rhodiola* species were separated more clearly compared to the scores plot of the whole region.

*R. crenulata* and *R. quadrifida* were also separated from the rest of the species. However, in this model they were clustered together. This suggests that there is no crucial metabolomic difference between them in the aromatic region. At this point it was considered important to visually inspect the spectra of the BRM's and detect any differences that might be lost with the integration of the data. *R. crenulata* BRM has an additional quartet at 6 ppm not detected in the rest of the species. This quartet can also be found in all the other *R. crenulata* samples investigated (figures not shown).

Therefore, an effective separation between *R. crenulata* and *R. quadrifida* samples can be accomplished by combining the PCA results with the detection of the additional peaks on the ^1^H-NMR spectra only present in *R. crenulata* samples between 5 and 6 ppm.

**Figure 5:**
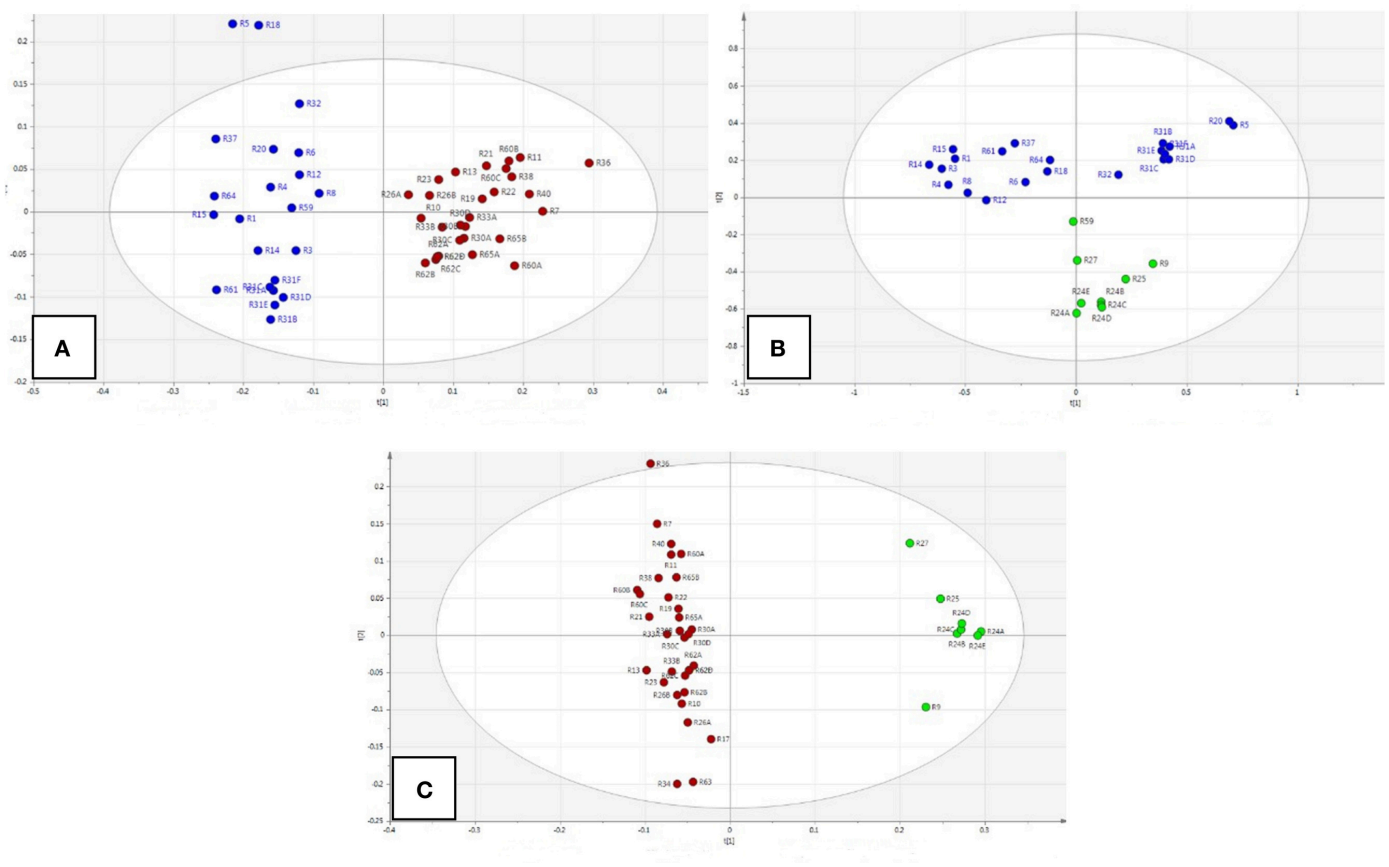
**Score plots of group comparison between *Rhodiola* species**. **(A)** *R. crenulata* (red) with other *Rhodiola* spp. (blue); **(B)** *R. rosea* (green) with other *Rhodiola* spp. (blue); **(C)** *R. crenulata* (red) with *R. rosea* (green).

We also studied the group-pair comparisons in PCA model with Par scaling (Figure 5). The score plots showed that *Rhodiola* species separated well (A: *R. crenulata* with *other Rhodiola species*; B: *R. rosea with other Rhodiola species;* C: *R. crenulata* with *R. rosea*).

The main differences were between δ 7.5–7.3 ppm (PC1) and δ 7.0–6.8 ppm (PC2). The chemical shift of the main variable metabolites were mainly rosavin, rosarin, and cinnamyl alcohol derivatives.

The metabolites detected were elucidated by the analyses of the ^1^H-NMR spectra as well as the comparison with the reference compounds, together with the in-house NMR chemical shift database (Mudge et al., 2013; Luo et al., 2015). The summary of the assignment of ^1^H-NMR spectral peaks obtained from the *R. rosea, R. crenulata*, and *R. sachalinensis* BRM extracts are provided in Supplement (S3).

### HPTLC analysis

**Figure 6:**
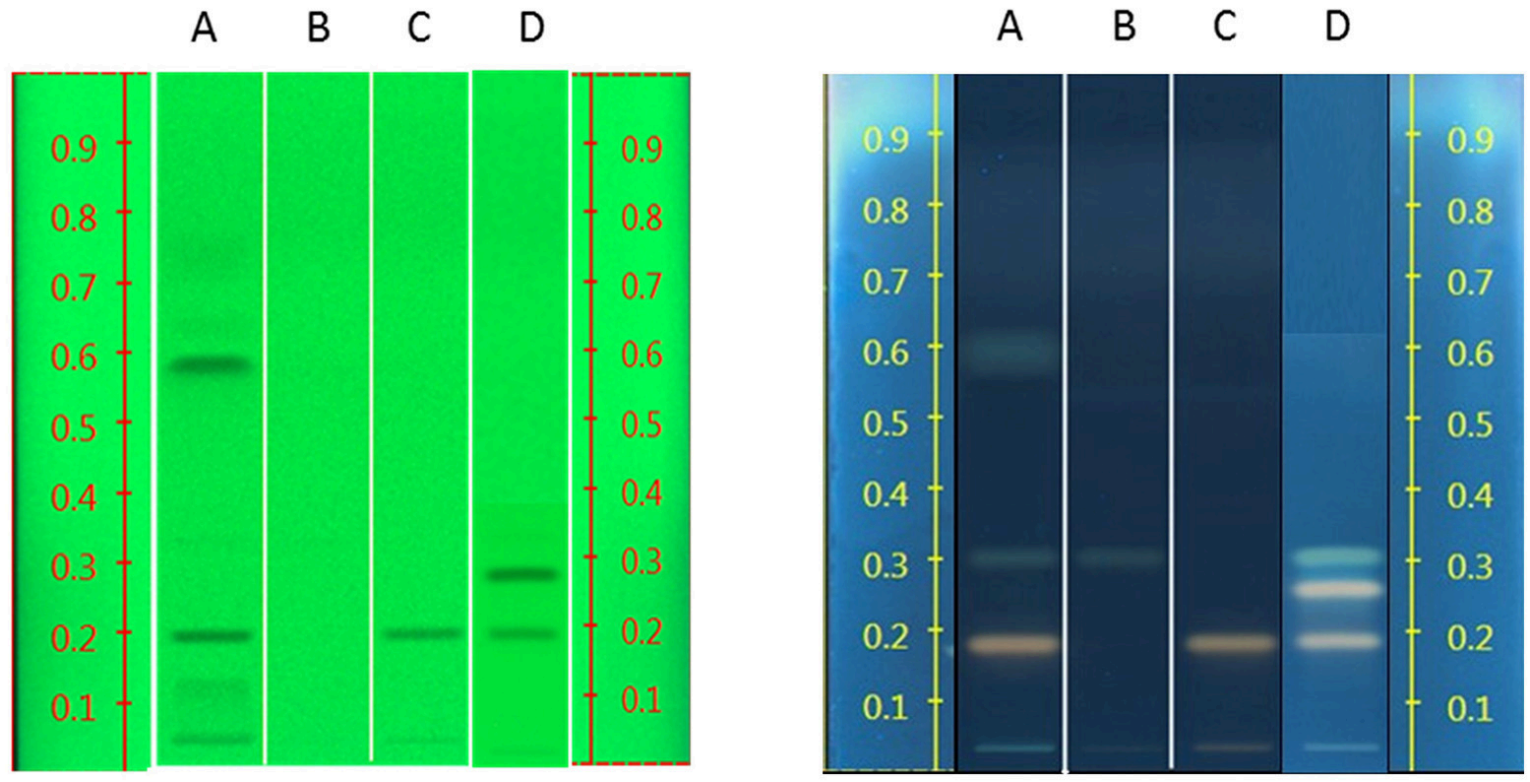
**Left: HPTLC results of standard compounds under UV 254 nm (rosavin Rf = 0.19, rosarin Rf = 0.26, gallic acid Rf = 0.58); Right: HPTLC results of standard compounds under UV 366 nm, after derivatisation with sulfuric acid (rosavin Rf = 0.19, rosarin Rf = 0.26, salidroside Rf = 0.31, gallic acid Rf = 0.58, tyrosol Rf = 0.76)**.

The band position and visibility of the standards rosavin, rosarin, and salidroside (Figure 6) appear with characteristic colors and increasing retention factors (Rfs) 0.19, 0.26, and 0.31, respectively. Under UV light 254 nm, salidroside is not visible. Under 366 nm, after derivitisation with sulfuric acid, rosavin and rosarin appear as pale pink bands and salidroside as a green one.

Gallic acid shows good visibility under UV 254 nm, while it is not easily detected under UV 366 nm at a dark blue back-round. Tyrosol is visible in 254 nm but less clear in 366 nm.

**Figure 7:**
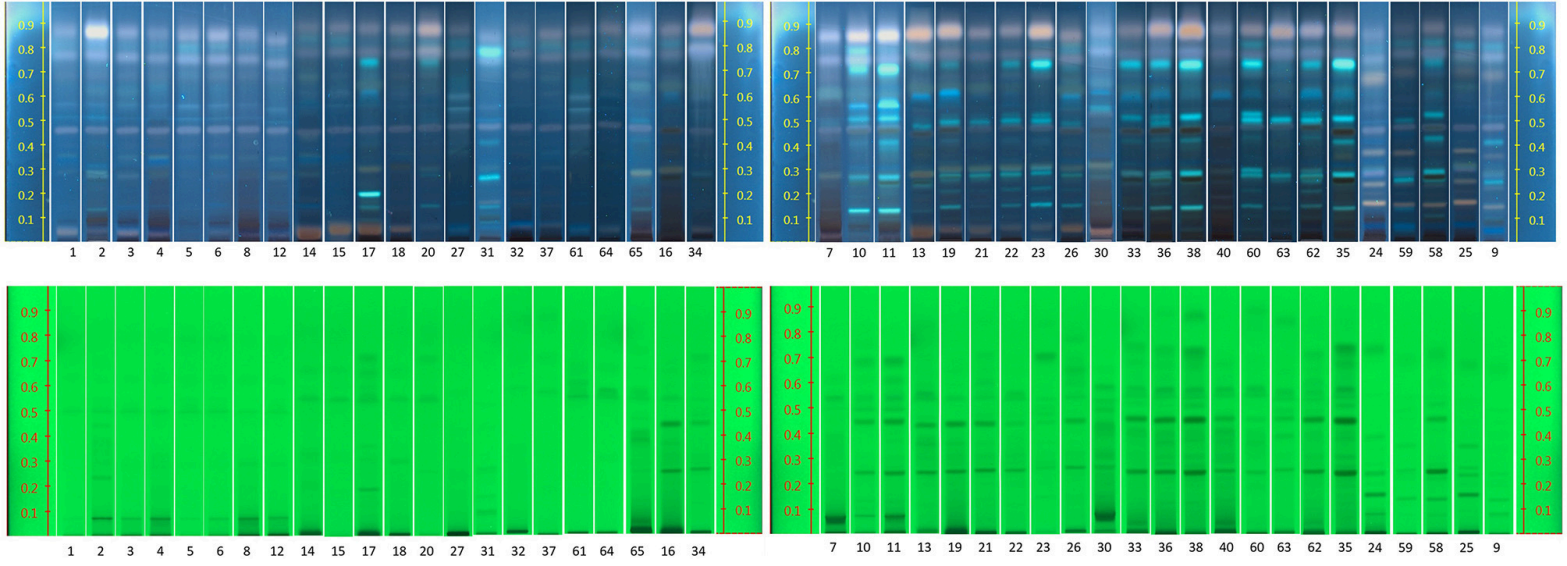
**HPTLC results for all *Rhodiola* market samples, mobile phase [Ethylacetate, methanol, water, formic acid (77:13:10:2)]**.

The raw plant material obtained from the market was also studied by our HPTLC method (list of samples in Supplement S4). Under UV 254 nm (Figure 7), there were two obvious bands among these samples (Rf = 0.27 and 0.48). However, due to lack of reference standards, their identity remains unknown. Further studies need to be conducted using NMR and LC-MS. The majority of the samples investigated contained concentrations of tyrosol similar to the standard raw material used (R24, R30, and R31). Samples R1–R6 contained lower levels of this compound possibly due to their longer storage time. Therefore, tyrosol could be considered as a marker to study duration of *Rhodiola* storage. It was also found that only five samples (R9, R25, R58, R59, and R24) contained high levels of rosavin, which turned out to be the ones from *R. rosea*. Moreover, this result can also be verified by the NMR results (Figure 5). However, it is not evident whether there is adulteration of *R. sachalinensis* in *R. rosea* since their metabolites are similar.

Under UV 366 nm after derivatisation eight samples (R1, R5, R6, R15, R27, R32, R61, and R64) had a low concentration of salidroside (Rf = 0.31). These samples could have been kept for a long time after collection and the salidroside content could have decreased due to lack of a good storage environment.

Combining the results of HPTLC and ^1^H-NMR multivariate statistical analysis, we also analyzed the adulteration rate among all the market samples (Supplement, S4).

Thirty percent of the *Rhodiola* samples collected from the market were not, as declared on the label, i.e., either *R. rosea* or *R. crenulata*. Some *R. rosea* samples were also being sold as *R. crenulata*. 47.7% of raw material samples were not labeled properly and their species information were not clearly illustrated to customers. This highlights a clear lack of proper local government policies and good quality control strategies.

According to our study, different *Rhodiola* species (including *R. rosea* and *R. crenulata)* can be found in the Chinese market. However, they are neither sold separately nor well-identified. Therefore, there is a high potential of adulteration and substitution among these species.

### Qualitative and quantitative analysis of mixtures

Since in the value chain, mixing of batches and, therefore, potentially also of species, is of major concern, the possibility of qualitatively and quantitatively detecting plant mixtures was also investigated. The additional species chosen for this study was *R. crenulata* which is considered to be the most common adulterant of *R. rosea*. The selected BRMs were weighed individually in different proportions and then added together in an Eppendorf reaction tube. The rest of the sample preparation was identical to the methodology for the ^1^H-NMR spectroscopy. The samples were renamed as seen in Table 4. After the acquisition of the spectra, they were baseline and phase corrected and zeroed to the TMS peak in Topspin 3.2.

**Table 4:** **^1^H-NMR-based detection of plant mixtures by**.

**Sample name**	**Mg of *R. rosea* BRM**	**Mg of *R. crenulata* BRM**
RR100	100	00
RR80RC20	80	20
RR60RC40	60	40
RR40RC60	40	60
RR20RC80	20	80
RC100	00	100

**Figure 8:**
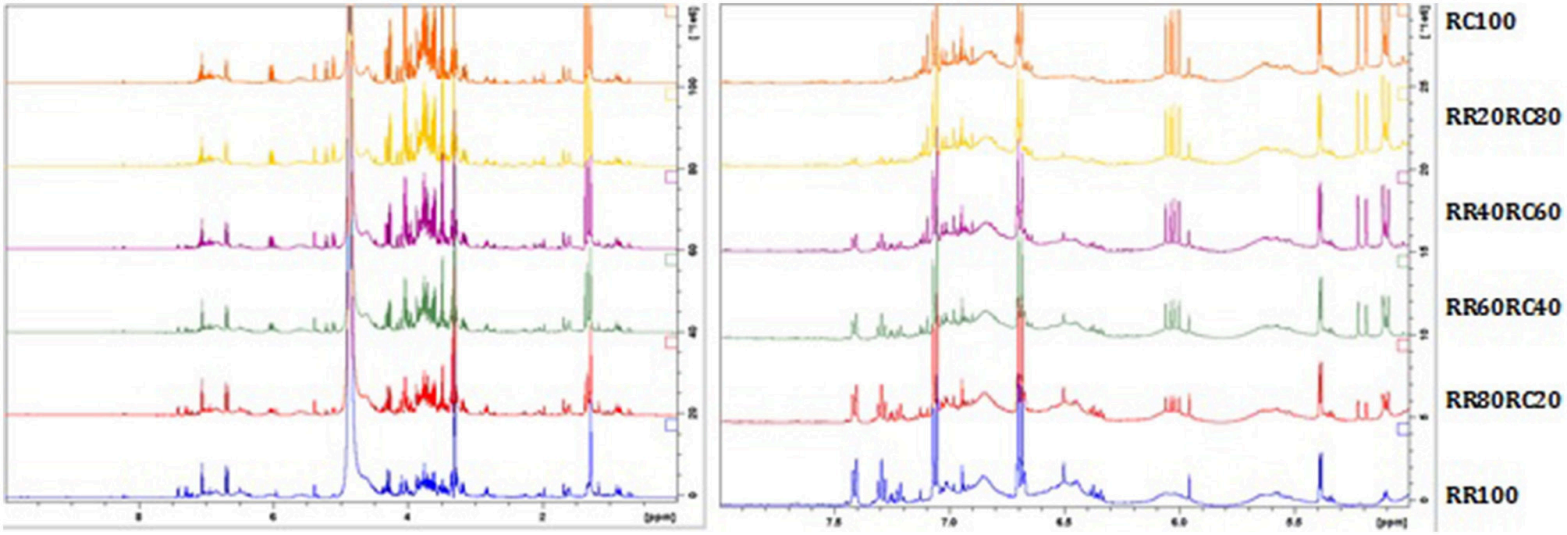
**^1^H-NMR spectra of the whole region (left) and the aromatic region (right) of the *R. rosea* and *R. crenulata* mixtures**.

In all samples, the salidroside peak intensity remains almost the same since this constituent is present in both species. The peaks of rosavin are gradually decreasing with the addition of *R. crenulata*, whereas the characteristic quartet at 6 ppm due to the presence of an unknown compound is increasing with the addition of *R. crenulata* and it is not detected in *R. rosea* at all (Figure 8).

**Figure 9:**
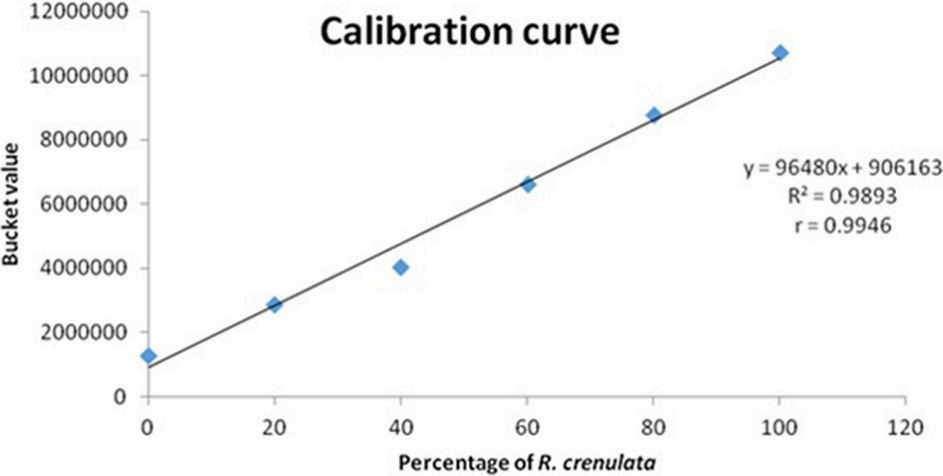
**Calibration curve showing the bucket value of the peak vs. the percentage of *R. crenulata* within a mixture of *R. crenulata* and *R. rosea***.

The acquired spectra were bucketed using Amix and only focused on this region (6 ppm). When the whole quartet was integrated into a single bucket, the observed increase of its intensity was not adequately represented. Therefore, the bucket size used changed to 0.002 ppm and only incorporated the first peak of the quartet (6.0028–6.0048 ppm). The buckets obtained from Amix were transferred into Excel, where the relationship between the bucket value and the percentage of *R. crenulata* in the mixture was expressed graphically as a calibration curve. The bucket value of the respective peak is increasing in a linear mode (Figure 9).

Similar results can also be obtained with HPTLC analysis. The HPTLC fingerprints produced consist of the over-spotted BRM extracts in different volumes as seen in Table 5. The final volume applied was 5 μl.

**Table 5:** **Sample preparation for the detection of plant mixtures by HPTLC**.

*R. rosea* 100%	RR100	*R. rosea* BRM	5 μL
*R. rosea* 80% and *R. crenulata* 20%	RR80	*R. rosea* BRM	4 μL
		*R. crenulata* BRM	1 μL
*R. rosea* 60% and *R. crenulata* 40%	RR60	*R. rosea* BRM	3 μL
		*R. crenulata* BRM	2 μL
*R. rosea* 40% and *R. crenulata* 60%	RR40	*R. rosea* BRM	2 μL
		*R. crenulata* BRM	3 μL
*R. rosea* 20% and *R. crenulata* 80%	RR20	*R. rosea* BRM	1 μL
		*R. crenulata* BRM	4 μL
*R. crenulata* 100%	RC100	*R. crenulata* BRM	5 μL

**Figure 10:**
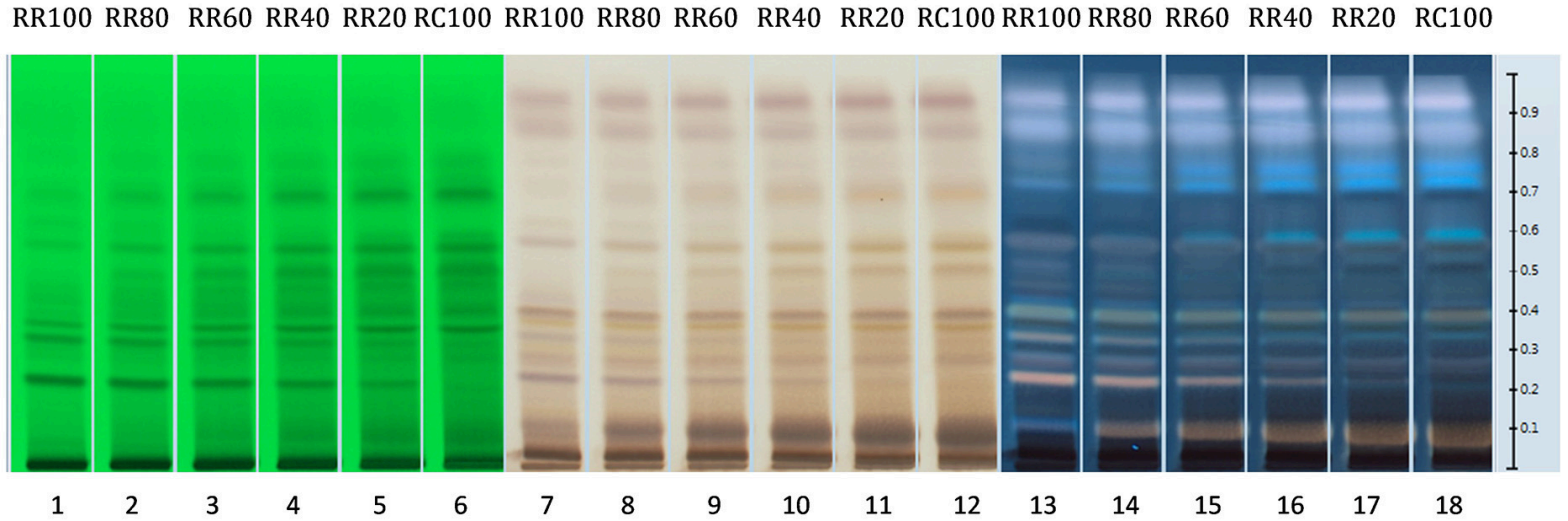
**HPTLC fingerprints of all *R. rosea* and *R. crenulata* mixtures under UV 254 nm (tracks 1–6), white light and SAR (tracks 7–12), and UV 366 nm and SAR (tracks 13–18)**.

As seen in Figure 10, when the loading volume of the *R. rosea* decreases, the representative markers of this species (rosavin and rosarin) decrease as well. However, the band for salidroside, (since it occurs in both species) remains almost the same.

By gradually increasing the *R. crenulata* proportion, several bands gradually appear above salidroside that could potentially be used as markers for the qualitative and semi-quantitative HPTLC analysis of mixtures of these two *Rhodiola* species. Further work needs to be carried out to determine the identity and species-specificity of these compounds.

## Conclusions

This study provided a method for distinguishing five different species of *Rhodiola* and suggests possible methods for quantifying different species within mixtures. The metabolomic and phytochemical differences between these different species has been demonstrated through NMR spectroscopy and HPTLC analysis. Species represented with only a small number of samples will need further investigation in order to accurately define their chemical characteristics.

There is a need to study the links between producers and consumers especially when in trans-national trade and re-enforce the hypothesis that poor quality and adulterated products can be products of poorly governed value chains, particularly at the early stages of supply. Moreover, through the establishment of well-controlled and well-managed value chains it is possible to better prevent accidental or deliberate contamination and adulteration from occurring.

## Author contributions

AB, main author and collector of samples in China, assisted with multivariate analysis of NMR data, responsible for contribution toward discussions and conclusions. CG, Responsible for HPTLC analysis and writing the methods, results, and part of the discussion for HPTLC. LZ, Responsible for NMR analysis and writing of the methods, results and part of the discussion relating to NMR. SL, Responsible for authentication of Chinese *Rhodiola* specimens and DNA analysis shown in supplementary data. MH, Principle investigator and overall director of the project, played a major role in the writing of the introduction and conclusions. All authors proof read manuscript and made contributions to the final version.

### Conflict of interest statement

The authors declare that the research was conducted in the absence of any commercial or financial relationships that could be construed as a potential conflict of interest.

## Abbreviations

TCM: Traditional Chinese Medicine
NMR: Nuclear magnetic resonance
HPTLC: High performance thin layer chromatography
spp: Species.

## References

[B1] Alm T. (2004). *Ethnobotany of Rhodiola rosea* (Crassulaceae) in Norway. Sida Contrib. Bot. 21, 321–344.

[B2] Aslanyan G. Amroyan E. Gabrielyan E. Nylander M. Wikman G. Panossian A. (2010). Double-blind, placebo-controlled, randomised study of single dose effects of ADAPT-232 on cognitive functions. Phytomedicine 17, 494–499. 10.1016/j.phymed.2010.02.005 20374974

[B3] Booker A. Frommenwiler D. Johnston D. Umealajekwu C. Reich E. Heinrich M. (2014). Chemical variability along the value chains of turmeric (Curcuma longa): a comparison of nuclear magnetic resonance spectroscopy and high performance thin layer chromatography. J. Ethnopharmacol. 152, 292–301. 10.1016/j.jep.2013.12.042 24417868

[B4] Booker A. Jalil B. Frommenwiler D. Reich E. Zhai L. Kulic Z.. (2015). The authenticity and quality of Rhodiola rosea products. Phytomedicine 23, 754–762. 10.1016/j.phymed.2015.10.006 26626192

[B5] Brown R. P. Gerbarg P. L. Ramazanov Z. (2002). *Rhodiola rosea*: A phytomedicinal overview. HerbalGram 56, 40–52.

[B6] Chinese Pharmacopoeia (2010). State Pharmacopoeia Committee. Pharmacopoeia of the People's Republic of China Part I. Beijing: China Medical Science Press.

[B7] Committee on Herbal Medicinal Products (2012). Community Herbal Monograph on Rhodiola rosea L., rhizoma et radix. EMA/HMPC/232091/2011. European Medicines Agency, 1–5.

[B8] Fan W. Tezuka Y. Ni K. M. Kadota S. (2001). Prolyl endopeptidase inhibitors from the underground part of *Rhodiola sachalinensis*. Chem. Pharm. Bull. (Tokyo) 49, 396–401. 10.1248/cpb.49.396 11310664

[B9] Galambosi B. (2006). Demand and availability of *Rhodiola rosea* raw material, in Medicinal and Aromatic Plants: Agricultural, Commercial, Ecological, Legal, Pharmacological and Social Aspects, Vol. 17, Wageningen UR Frontis Series, eds Bogers R. J. Craker L. E. Lange D. (Springer), 223–236.

[B10] Jiang M. Wang C. Zhang Y. Feng Y. Wang Y. Zhu Y. (2014). Sparse partial-least-squares discriminant analysis for different geographical origins of *Salvia miltiorrhiza* by (1) H-NMR-based metabolomics. Phytochem. Anal. 25, 50–58. 10.1002/pca.2461 23868756

[B11] Li T. Zhang H. (2008). Identification and comparative determination of rhodionin in traditional tibetan medicinal plants of fourteen Rhodiola species by high-performance liquid chromatography-photodiode array detection and electrospray ionization-mass spectrometry. Chem. Pharm. Bull. (Tokyo) 56, 807–814. 10.1248/cpb.56.807 18520085

[B12] Li Z. Y. Zhang Z. Z. Du G. H. Qin X. M. (2014). Comparative analysis of Danggui and European Danggui using nuclear magnetic resonance-based metabolic fingerprinting. J. Pharm. Biomed. Anal. 103c, 44–51. 10.1016/j.jpba.2014.10.028 25462119

[B13] Lu J. Lan X. (2013). The characteristics of the rare and endangered tibetan medicinal plant resources in Shannan Region (in Chinese). J. Nat. Resour. 28, 1977–1987. 10.11849/zrzyxb.2013.11.014

[B14] Luo X. Wang X. J. Li S. P. Zhang Q. Zhao Y. W. Huang W.-z. (2015). [Simultaneously preparation of grams of high purity tyrosol, crenulatin and salidroside from *Rhodiola crenulata*]. Zhongguo Zhong Yao Za Zhi, 40, 1300–1304. 26281551

[B15] Ma G. Li W. Dou D. Chang X. Bai H. Satou T.. (2006). Rhodiolosides A-E, monoterpene glycosides from *Rhodiola rosea*. Chem. Pharm. Bull. (Tokyo) 54, 1229–1233. 10.1248/cpb.54.1229 16880679

[B16] Mudge E. Lopes-Lutz D. Brown P. N. Schieber A. (2013). Purification of Phenylalkanoids and monoterpene glycosides from *Rhodiola rosea* L. roots by high-speed counter-current chromatography. Phytochem. Anal. 24, 129–134. 10.1002/pca.2391 22811209

[B17] Nakamura S. Li X. Matsuda H. Yoshikawa M. (2008). Bioactive constituents from Chinese natural medicines. XXVIII. Chemical structures of acyclic alcohol glycosides from the roots of Rhodiola crenulata. Chem. Pharm. Bull. (Tokyo) 56, 536–540. 10.1248/cpb.56.536 18379104

[B18] Nakamura S. Li X. Matsuda H. Ninomiya K. Morikawa T. Yamaguti K.. (2007). Bioactive constituents from Chinese natural medicines. XXVI. Chemical structures and hepatoprotective effects of constituents from roots of Rhodiola sachalinensis. Chem. Pharm. Bull. (Tokyo) 55, 1505–1511. 10.1248/cpb.55.1505 17917296

[B19] Panossian A. Hamm R. Kadioglu O. Wikman G. Efferth T. (2013). Synergy and antagonism of active constituents of ADAPT-232 on transcriptional level of metabolic regulation of isolated neuroglial cells. Front. Neurosci. 7:16. 10.3389/fnins.2013.00016 23430930 PMC3576868

[B20] Panossian A. Wikman G. Sarris J. (2010). Rosenroot (*Rhodiola rosea*): traditional use, chemical composition, pharmacology and clinical efficacy. Phytomedicine 17, 481–493. 10.1016/j.phymed.2010.02.002 20378318

[B21] Reich E. Schibli A. DeBatt A. (2008). Validation of high-performance thin-layer chromatographic methods for the identification of botanicals in a cGMP environment. J. AOAC Int. 91, 13–20. 18376581 PMC2662610

[B22] Sarris J. Panossian A. Schweitzer I. Stough C. Scholey A. (2011). Herbal medicine for depression, anxiety and insomnia: a review of psychopharmacology and clinical evidence. Eur. Neuropsychopharmacol. 21, 841–860. 10.1016/j.euroneuro.2011.04.002 21601431

[B23] Shikov A. N. Pozharitskaya O. N. Makarov V. G. Wagner H. Verpoorte R. Heinrich M. (2014). Medicinal Plants of the Russian Pharmacopoeia; their history and applications. J. Ethnopharmacol. 154, 481–536. 10.1016/j.jep.2014.04.007 24742754

[B24] Simmler C. Napolitano J. G. McAlpine J. B. Chen S. N. Pauli G. F. (2014). Universal quantitative NMR analysis of complex natural samples. Curr. Opin. Biotechnol. 25, 51–59. 10.1016/j.copbio.2013.08.004 24484881 PMC3912461

[B25] World Health Organization (2013). Plants, Medicinal, Volume 1, Medicinal Plants in Mongolia. WHO Western Pacific Region Publication.

[B26] Xia T. Chen S. Chen S. Ge X. (2005). Genetic variation within and among populations of *Rhodiola* alsia (Crassulaceae) native to the Tibetan Plateau as detected by ISSR markers. Biochem. Genet. 43, 87–101. 10.1007/s10528-005-1502-5 15932059

[B27] Xin T. Li X. Yao H. Lin Y. Ma X. Cheng R.. (2015). Survey of commercial *Rhodiola* products revealed species diversity and potential safety issues. Sci. Rep. 5, 8337. 10.1038/srep08337 25661009 PMC4321177

[B28] Yousef G. G. Grace M. H. Cheng D. M. Belolipov I. V. Raskin I. Lila M. A. (2006). Comparative phytochemical characterization of three Rhodiola species. Phytochemistry 67, 2380–2391. 10.1016/j.phytochem.2006.07.026 16956631

[B29] Zhi H. J. Qin X. M. Sun H. F. Zhang L. Z. Guo X. Q. Li Z. Y. (2012). Metabolic fingerprinting of *Tussilago farfara* L. using (1)H-NMR spectroscopy and multivariate data analysis. Phytochem. Anal. 23, 492–501. 10.1002/pca.2346 22371211

[B30] Zhu L. Lou A. (2010). Mating system and pollination biology of a high-mountain perennial plant, *Rhodiola* dumulosa (Crassulaceae). J. Plant Ecol. 3, 219–227. 10.1093/jpe/rtq024

