# Supplementary material for: From Traditional Resource to Global Commodities:—A Comparison of Rhodiola Species Using NMR Spectroscopy—Metabolomics and HPTLC
Source: Front Pharmacol. 2016 Aug 29;7:254. doi: 10.3389/fphar.2016.00254 (PMC5002433; doi:10.3389/fphar.2016.00254)
Supplement: Supplementary file 1 [file DataSheet1.PDF]

S1 Sample Information

A) Samples collection information of *Rhodiola* spp. (for Figures 2, 4)

**Sample No**	**Sample name**	**Scientific name**	**Plant part**	**Provider**	**Place of origin**	**Harvest Year**	**Additional info**
S1	Mattmark 2009	*R.rosea*	Rhizomes	Agroscope	Mattmark, Switzerland	2009	Mix of several individuals propagated by seeds from the wild population of Mattmark
S2	M1 2010	*R.rosea*	Rhizomes	Agroscope	Mattmark, Switzerland	2010	Single individual propagated vegetatively from the wild population of Mattmark
S3	M2 2010	*R.rosea*	Rhizomes	Agroscope	Mattmark, Switzerland	2010	Single individual propagated vegetatively from the wild population of Mattmark
S4	M3 2010	*R.rosea*	Rhizomes	Agroscope	Mattmark, Switzerland	2010	Single individual propagated vegetatively from the wild population of Mattmark
S5	C1 2010	*R.rosea*	Rhizomes	Agroscope	Piano	2010	Single individual propagated vegetatively from the wild

					Canali, Switzerland		population of Carrasino (Ticino)
S6	JB Geneve 2010	*R.rosea*	Rhizomes	Agroscope	Botanical garden of Geneva, Switzerland	2010	Single individual propagated vegetatively from the botanical garden of Geneva
S7	JB Natn 2010	*R.rosea*	Rhizomes	Agroscope	Botanical garden, not specified	2010	Single individual propagated vegetatively from a botanical garden
S8	Jena 2010	*R.rosea*	Rhizomes	Agroscope	Botanical garden of Jena, Germany	2010	Single individual propagated by seeds from the botanical garden of Jena, Germany
S9	Jellito	*R.rosea*	Rhizomes	Agroscope	Not	2010	Grown from seeds purchased from "Jellito" seed supplier

	2010				specified		
S10	Norway 2010 F	*R.rosea*	Rhizomes	Agroscope	Norway	2010	Female plant from Dr. Bertalan Galambosi
S11	Norway 2010 M	*R.rosea*	Rhizomes	Agroscope	Norway	2010	Male plant from Dr. Bertalan Galambosi
S12	M2 2012	*R.rosea*	Rhizomes	Agroscope	Mattmark, Switzerland	2012	Single individual propagated vegetatively from the wild population of Mattmark
S13	M6 2012	*R.rosea*	Rhizomes	Agroscope	Mattmark, Switzerland	2012	Single individual propagated vegetatively from the wild population of Mattmark
S14	M8 2012	*R.rosea*	Rhizomes	Agroscope	Mattmark, Switzerland	2012	Single individual propagated vegetatively from the wild population of Mattmark
S15	M15 2012	*R.rosea*	Rhizomes	Agroscope	Mattmark, Switzerland	2012	Single individual propagated vegetatively from the wild population of Mattmark

S16	N1 2012	*R.rosea*	Rhizomes	Agroscope	Nomnom, Switzerland	2012	Single individual propagated vegetatively from the wild population of Mattmark
S17	Jena 2013	*R.rosea*	Rhizomes	Agroscope	Botanical garden of Jena, Germany	2013	Single individual propagated by seeds from the botanical garden of Jena, Germany
S18	JB Geneve 2013	*R.rosea*	Rhizomes	Agroscope	Botanical garden of Geneva, Switzerland	2013	Single individual propagated vegetatively from the botanical garden of Geneva
S19	R57	*R.rosea*	Root	ChromaDex	Not mentioned	Not specified	Extract, collected in London, Jan. 2015
S20	R1	*R.rosea*	Root	Dong Ma Dao	Xinjiang, China	Not specified	Collected in Zhangjiakou, Hebei, Oct. 2014

				pharmacy			
S21	R24	*R.rosea*	Root	Schwabe Pharm Co.	Germany	Not specified	Collected in London, Nov. 2014
S22	R37	*R.rosea*	Root	Bozhou Changsheng crude drug root Co., Ltd	Tibet	Not specified	Collected in Beijing, Dec. 2014
S23	R31	*R.sachalinensis*	Powdered roots & rhizomes	NICPBP	NE of China	Not specified	Collected in Beijing, Nov. 2014
S24	R03	*R.sachalinensis*	Root	Retailer, unspecified	Baishan, Jilin, China	Not specified	Collected in Jingyu, Jilin, Oct.2014
S25	R09	*R.sachalinensis*	Root	Internet,	Tonghua,	Not specified	Collected in Ji'an, Jilin, Oct.2014

				unspecified	Jilin, China		
S26	R30	*R.crenulata*	Powdered roots & rhizomes	N.I.C.P.B.P.	Tibet	Not specified	Collected in Beijing, Nov. 2014
S27	R04	*R.crenulata*	Root	Retailer, unspecified	Naqu, Tibet	Not specified	Collected in Ge'ermu, Qinghai, Oct.2014
S28	R13	*R.crenulata*	Root	BoZhou TCM drug market	Tibet	Not specified	Collected in Bozhou, Anhui, Oct.2014
S29	R14	*R.crenulata*	Root	Retailer, unspecified	Naqu, Tibet	Not specified	Collected in Ge'ermu, Qinghai, Oct.2014
S30	R35	*R.crenulata*	Root	Qing Ping TCM drug	Tonghua, Jilin, China	Not specified	Collected in Guangzhou, Guangdong, Dec.2014

				market			
S31	R40	*R.crenulata*	Root	Hong Kong Dongxigtang technology Co., Ltd	Tibet	Not specified	Collected in Hong Kong, Dec.2014
S32	Shikov 2015	*R.quadrifida*	Rhizomes	Prof. Alexander Shikov	Korgon ridge, Altai, Russia	2014	Sent by Prof. Alexander Shikov, May 2015
S33	R10	*R.quadrifida*	Root	Qing Ping TCM drug market	Tibet	Not specified	Collected in Guangzhou, Guangdong, Oct.2014
S34	R32	*R.quadrifida*	Root	Qing Ping TCM drug	Tibet	Not specified	Collected in Guangzhou, Guangdong, Dec.2014

				market			
S35	R34	*R.quadrifida*	Root	Qing Ping TCM drug market	Sichuan	Not specified	Collected in Guangzhou, Guangdong, Dec.2014
S36	R56	*R.quadrifida*	Powdered root	Retailer, unspecified	Russia	Not specified	Collected in Russia, Dec.2014
S37	0615-01	*R.crenulata*	Root	Dr. Anthony Booker	Garze, Sichuan	2015	Single individual, collected from the wild, June2015
S38	0615-02	*R.fastigiata*	Root and rhizome	Dr. Anthony Booker	Garze, Sichuan	2015	Single individual, collected from the wild, June2015
S39	0615-03	*R.crenulata*	Root and rhizome	Dr. Anthony Booker	Garze, Sichuan	2015	Single individual, collected from the wild, June2015
S40	0615-04	*R.crenulata*	Root and	Dr. Anthony	Garze,	2015	Single individual, collected from the wild, June2015

			rhizome	Booker	Sichuan		
S41	0615-05	*R.crenulata*	Stems	Dr. Anthony Booker	Garze, Sichuan	2015	Single individual, collected from the wild, June2015
S42	0615-06	*R.fastigiata*	Root	Dr. Anthony Booker	Garze, Sichuan	2015	Single individual, collected from the wild, June2015

B) Samples collection information of *Rhodiola* spp. (for Figures 5, 7)

No.	Trade Name	Rename	Scientific Name	Sample details	Channel	Origin details	Collection Place	Place of Origin	Collection Date
1	Hong Jing Tian	R1	*Rhodiola rosea*	Decoction pieces	Retailer	Dong Ma Dao Pharmacy	Zhangjiakou, Hebei	Xinjiang	Oct. 2014
2	Hong Jing Tian	R2	*Rhodiola rosea*	Crude drug root	Market	Bo Zhou TCM market	Bozhou, Anhui	Tibet	Oct. 2014
3	Ku Ye Hong Jing Tian	R3	*Rhodiola sachalinensis*	Crude drug root	Retailer	Not mentioned	Jingyu, Jilin	Baishan, Jilin	Oct. 2014
4	Da Hua Hong Jing Tian	R4	*Rhodiola crenulata*	Crude drug root	Retailer	Not mentioned	Ge'ermu, Qinghai	Naqu, Tibet	Oct. 2014
5	Hong Jing Tian	R5	*Rhodiola rosea*	Crude drug root	Market	An Guo TCM market	Anguo, Hebei	Tibet	Oct. 2014

No.	Trade Name	Rename	Scientific Name	Sample details	Channel	Origin details	Collection Place	Place of Origin	Collection Date
6	Ku Ye Hong Jing Tian	R6	*Rhodiola sachalinensis*	Crude drug root	Market	Bai Shan TCM market	Baishan, Jilin	Baishan, Jilin	Oct. 2014
7	Hong Jing Tian	R7	*Rhodiola rosea*	Decoction pieces	Retailer	Tong Ren Tang	Shijiazhuang, Hebei	Linzhi, Tibet	Oct. 2014
8	Hong Jing Tian	R8	*Rhodiola rosea*	Crude drug root	Internet	Not mentioned	Ji’an, Jilin	Tonghua, Jilin	Oct. 2014
9	Ku Ye Hong Jing Tian	R9	*Rhodiola sachalinensis*	Crude drug root	Internet	Not mentioned	Ji’an, Jilin	Tonghua, Jilin	Oct. 2014
10	Si Lie Hong Jing Tian	R10	*Rhodiola quadrifida*	Crude drug root	Market	Qing Ping TCM drug market	Guangzhou, Guangdong	Tibet	Oct. 2014
11	Hong Jing Tian	R11	*Rhodiola rosea*	Crude drug root	Market	Qing Ping TCM drug market	Guangzhou, Guangdong	Tibet	Oct. 2014

No.	Trade Name	Rename	Scientific Name	Sample details	Channel	Origin details	Collection Place	Place of Origin	Collection Date
12	Da Hua Hong Jing Tian	R12	*Rhodiola crenulata*	Crude drug root	Market	Qing Ping TCM drug market	Guangzhou, Guangdong	Sichuan	Oct. 2014
13	Da Hua Hong Jing Tian	R13	*Rhodiola crenulata*	Crude drug root	Market	Bo Zhou TCM drug market	Bozhou, Anhui	Tibet	Oct. 2014
14	Da Hua Hong Jing Tian	R14	*Rhodiola crenulata*	Crude drug root	Retailer	Not mentioned	Ge'ermu, Qinghai	Naqu, Tibet	Oct. 2014
15	Hong Jing Tian	R15	*Rhodiola* (unspecified)	Decoction pieces	Retailer	Jin Xing Hang	Hong Kong	Tibet (unverified)	Oct. 28, 2014
16	Hong Jing Tian	R16	*Rhodiola* (unspecified)	Decoction pieces	Retailer	Bao Xing Hang	Hong Kong	Not mentioned	Oct. 28, 2014
17	Hong Jing Tian	R17	*Rhodiola* (unspecified)	Decoction pieces	Retailer	Tai Tung Trading Co	Hong Kong	Tibet (unverified)	Oct. 28, 2014

No.	Trade Name	Rename	Scientific Name	Sample details	Channel	Origin details	Collection Place	Place of Origin	Collection Date
18	Hong Jing Tian	R18	*Rhodiola* (unspecified)	Decoction pieces	Retailer	Sun Lap Trading Co	Hong Kong	Tibet (unverified)	Oct. 28, 2014
19	Hong Jing Tian	R19	*Rhodiola* (unspecified)	Decoction pieces	Retailer	Wing Sang Wholesale Ltd.	Hong Kong	Tibet (unverified)	Oct. 28, 2014
20	Hong Jing Tian	R20	*Rhodiola* (unspecified)	Crude drug root	Retailer	Ceng Fu Ji Yao Hang	Hong Kong	Tibet (unverified)	Oct. 28, 2014
21	Hong Jing Tian	R21	*Rhodiola* (unspecified)	Crude drug root	Retailer	Hang Hing Medicine, Co., Ltd.	Hong Kong	Tibet (unverified)	Oct. 28, 2014
22	Hong Jing Tian	R22	*Rhodiola* (unspecified)	Crude drug root	Retailer	Xin Sheng Yao Ye, co., Ltd.	Hong Kong	Tibet (unverified)	Oct. 28, 2014
23	Hong Jing Tian	R23	*Rhodiola*	Crude	Retailer	Hang Hing	Hong Kong	Sichuan	Oct. 28,

No.	Trade Name	Rename	Scientific Name	Sample details	Channel	Origin details	Collection Place	Place of Origin	Collection Date
			(unspecified)	drug root		Medicine Co.,		(unverified)	2014
24	Hong Jing Tian	R24	*Rhodiola rosea*	Crude drug root	Institute	Schwabe Pharm Co.,	London	Germany	Nov. 2014
25	Hong Jing Tian	R25	*Rhodiola* (unspecified)	Crude drug root	Institute	Not mentioned	London	Russia	Nov. 2014
26	Hong Jing Tian	R26	*Rhodiola* (unspecified)	Crude drug root	Retailer	Not mentioned	London	Tibet	Nov. 2014
27	Hong Jing Tian	R27	*Rhodiola* (unspecified)	Crude drug root	Retailer	Not mentioned	UK	Sichuan	Dec. 2014
30	Da Hua Hong Jing Tian	R30	*Rhodiola crenulata*	Root powder	Institute	NICPBP	Beijing	Tibet	Nov. 2014
31	Ku Ye (Gao	R31	*Rhodiola*	Root	Institute	NICPBP	Beijing	Northeast of	Nov. 2014

No.	Trade Name	Rename	Scientific Name	Sample details	Channel	Origin details	Collection Place	Place of Origin	Collection Date
	Shan) Hong Jing Tian		*sachalinensis*	powder				China	
32	Si Lie Hong Jing Tian	R32	*Rhodiola quadrifida*	Crude drug root	Market	Qing Ping TCM drug market	Guangzhou, Guangdong	Tibet	Dec. 2014
33	Hong Jing Tian	R33	*Rhodiola rosea*	Crude drug root	Market	Qing Ping TCM drug market	Guangzhou, Guangdong	Hunjiang, Jilin	Dec. 2014
34	Si Lie Hong Jing Tian	R34	*Rhodiola quadrifida*	Crude drug root	Market	Qing Ping TCM drug market	Guangzhou, Guangdong	,Sichuan	Dec. 2014
35	Hong Jing Tian	R35	*Rhodiola crenulata*	Crude drug root	Market	Qing Ping TCM drug market	Guangzhou, Guangdong	Tonghua, Jilin	Dec. 2014
36	Hong Jing Tian	R36	*Rhodiola* (unspecified)	Crude drug root	Internet	Tian Ling Crude drug root Co.	Suzhou, Jiangsu	Tibet	Dec. 2014

No.	Trade Name	Rename	Scientific Name	Sample details	Channel	Origin details	Collection Place	Place of Origin	Collection Date
37	Hong Jing Tian	R37	*Rhodiola rosea*	Crude drug root	Internet	Bozhou Changsheng Crude drug root Co., Ltd	Beijing	Tibet	Dec. 2014
38	Hong Jing Tian	R38	*Rhodiola* (unspecified)	Crude drug root	Internet	Beijing Sheng Shi Long PhaR. Co	Beijing	Tibet	Dec. 2014
40	Hong Jing Tian	R40	*Rhodiola crenulata*	Crude drug root	Retailer	Hong Kong Dongxingtang technology Co., Ltd	Hong Kong	Tibet	Dec, 2014
56	Красная щемка	R56	*Rhodiola quadrifida*	Tea pack	Retailer	Not mentioned	Russia	Russia	Dec.2014

No.	Trade Name	Rename	Scientific Name	Sample details	Channel	Origin details	Collection Place	Place of Origin	Collection Date
58	Rhodiola rosea	R58	*Rhodiola rosea*	Crude drug powder	Institute	Not mentioned	London	Not mentioned	Jan.2015
59	4# Hong Jing Tian	R59	*Rhodiola* (unspecified)	Crude drug root	Retailer	Li Xiong Ji	Hong Kong	Xinjiang, China	Jan.2015
60	3# Hong Jing Tian	R60	*Rhodiola* (unspecified)	Crude drug root	Retailer	Li Xiong Ji	Hong Kong	Tibet, China	Jan.2015
61	1# Hong Jing Tian	R61	*Rhodiola* (unspecified)	Crude drug root	Retailer	Li Xiong Ji	Hong Kong	Tibet, China	Jan.2015
62	2# Hong Jing Tian	R62	*Rhodiola* (unspecified)	Crude drug root	Retailer	Li Xiong Ji	Hong Kong	Tibet, China	Jan.2015
63	Hong Jing Tian	R63	*Rhodiola*	Crude	Market	He Hua Chi	London	Chengdu,	Jan.2015

No.	Trade Name	Rename	Scientific Name	Sample details	Channel	Origin details	Collection Place	Place of Origin	Collection Date
			(unspecified)	drug root		TCM market		China	
64	Hong Jing Tian	R64	*Rhodiola* (unspecified)	Crude drug root	Market	He Hua Chi TCM market	London	Chengdu, China	Jan.2015
65	Hong Jing Tian	R65	*Rhodiola* (unspecified)	Crude drug root	Market	He Hua Chi TCM market	London	Chengdu, China	Jan.2015

S2 DNA amplification

Table.1 Sequence length of the ITS2 and barcode of *Rhodiola* spp.

Species	Sequence length (bp)
Length in *R. crenulata* (bp)	247
Length in *R. rosea* (bp)	247
Length in *R. sachalinensis* (bp)	248
Length in other *Rhodiola* species (bp)	247-252
Aligned length (bp)	247

Table.2 Sequence characteristics of the ITS2 of *Rhodiola* spp.

Species	G+C content range (mean)
R. crenulata (%)	59.56
R. rosea (%)	55.74

R. sachalinensis (%)	55.74
Other *Rhodiola* species (%)	55.20-59.56 (57.68)

S3 Metabolite identification

Table.3 Assignments of ^{1}H-NMR spectral peaks obtained from R24, R30 and R31 methanolic extracts

No.	Metabolite	δ ^{1}H multiplicity (ppm)	Assigned with	Source
**Secondary metabolite**				
1	Tyrosal	^{1}H-NMR (500 MHz, CD_3OD) δ: 6.70 (2H, d, J=8.4 Hz, H-2, 6), 7.01 (2H, d, J=8.4 Hz, H-3, 5), 2.70 (2H, t, J=7.2 Hz, H-7), 3.67 (2H, t, J=7.2 Hz, H-8).	COSY, HSQC	R24, R30, R31
2	Salidroside	^{1}H-NMR (500MHz, CD_3OD) δ: 6.69 (2H, d, J=8.3 Hz, H-2, 6), 7.05 (2H, d, J=8.3 Hz, H-3, 5) , 2.82 (2H, t, J=7.4 Hz, H-7), 3.67 (2H, m, H-8); Glucose: 4.29 (1H, d, J=7.7 Hz, H-1′), 3.19 (1H, t, J=8.2 Hz, H-2′), 3.27-3.37 (3H, m, H-3′, 4′,5′), 3.87 (1H, d, J=10.8 Hz, H-6′), 4.02 (1H, d, J=9.3 Hz, H-6′).	COSY	R24, R30, R31
3*	Rosavin	^{1}H-NMR (500 MHz, CD_3OD) δ: 7.42 d (2 H, J = 7.5 Hz), 7.30 t (2 H, J = 7.5 Hz), 7.22 t (1 H, J = 7.5 Hz), 6.70 d (1 H, J = 16.5 Hz), 6.37 dt (1 H, J = 16.0, 6.0 Hz,), 4.51 ddd (1 H, J = 12.5, 6.5, 1.5 Hz), 4.31m (1 H), 4.37 d (1 H, J = 7.5	COSY	R24, R30

No.	Metabolite	δ ^{1}H multiplicity (ppm)	Assigned with	Source
		Hz), 4.34 d (1 H, J = 7.0 Hz), 4.11 dd (1 H, J = 11.0, 2.5 Hz), 3.87 dd (1 H, J = 12.5, 3.5 Hz), 3.80m (1 H), 3.74 dd (1 H, J = 11.5, 6.0 Hz), 3.61 dd (1 H, J = 11.0, 7.0 Hz), 3.53m (1 H), 3.52m (1 H), 3.46m (1 H), 3.36m (1 H), 3.24m (1 H).		
4*	Rosarin	^{1}H-NMR (500 MHz, CD_3OD) δ: 7.41 d (2 H, J = 7.5 Hz), 7.29 t (2 H, J = 7.5 Hz) 7.21 t (1 H, J=7.5Hz), 6.69 d (1 H, J=16.0Hz), 6.36 dt (1 H, J = 16.0, 6.0 Hz), 4.98 d (1 H, J=1.5Hz), 4.50 ddd (1 H, J = 12.5, 6.5, 1.5 Hz), 4.36 d (1 H, J = 7.5 Hz), 4.31 ddd (1 H, J = 12.5, 6.5, 1.5 Hz), 4.15 dd (1 H, J = 12.0, 2.5 Hz), 4.01 dd (1 H, J = 3.3, 1.5 Hz), 3.98 ddd (1H, J=5.0, 5.0, 3.5Hz), 3.83 dd (J = 6.0, 3.5Hz), 3.74 dd (1H, J = 11.5, 2.5Hz), 3.62m (2 H), 3.45m (1H), 3.34m (1H), 3.29m (1H), 3.22m (1H).	COSY	R24
5*	Rosin	^{1}H-NMR (500 MHz, CD_3OD) δ: 7.40 d (2 H, J = 7.5 Hz), 7.29 t (2 H, J = 7.5 Hz), 7.21 t (1 H, J = 7.5 Hz), 6.68 d (1 H, J = 16.1 Hz, H-7),6.36 dt (1 H, J = 16.1, 5.6 Hz), 4.52 ddd (1 H, J = 12.6, 6.5, 1.5 Hz), 4.36 d(1 H, J=7.5Hz), 4.32	COSY	R24

No.	Metabolite	δ ^{1}H multiplicity (ppm)	Assigned with	Source
		ddd (1 H, J = 12.6, 6.5, 1.5 Hz), 3.87 dd (1 H, J=12.6,2.5 Hz), 3.67 dd (1 H, J = 11.9, 5.6 Hz), 3.32–3.38m (4 H).		
6*	Crenulatin	^{1}H-NMR (500MHz, CD_3OD) δ: 1.34 (s), 1.37 (s), 4.36 (d, 7.2), 5.97 (m, J_{ab} = 17.6 Hz, J_{ac}= 11Hz, J_{ba}= 17.6 Hz, J_{ca}= 11Hz, 2-H_a, 1-H_b, 1-H_c).	COSY, HMBC, HSQC	R30
**Organic acid**				
7	Gallic acid	^{1}H-NMR (500MHz, CD_3OD) δ: 7.03 (2H, s, H-3, 5).	COSY	R24, R30, R31
8	Acetate	δ: 1.92 (s)	–	R24, R30, R31
**Glucose**				
9	β-glucose	δ: 4.26 (d, 8.0 Hz, 1-CH).	–	R24, R30, R31

No.	Metabolite	δ ^{1}H multiplicity (ppm)	Assigned with	Source
10	Fructose	δ: 4.10 (d, J=8.7)	–	R24, R30, R31
**Amino acid**				
11	Leucine	δ: 0.94 (t, J=5.9)	–	R24, R30, R31
12	Isoleucine	δ: 0.98 (d, J=7.0)	–	R24, R30, R31
13	Valine	δ: 1.05 (d, J=7.1)	–	R24, R30, R31
14	Alanine	δ: l,48 (d, J=7.3)	–	R24, R30, R31
15	Proline	δ: 2.06 (m)	–	R24, R30, R31

Table.6 Reproducibility of sample preparation and NMR measurements of five different samples prepared independently from the same sample (R24 *R. rosea*); Particular compound was used R 30 *R. crenulata*.

No.	Metabolite	$\overline{x}$	SD (n=5)	RSD (%)
1	Tyrosal	0.064340425	0.003871713	6.017543221
2	Salidroside	0.149687767	0.008947747	5.977607381
3	Rosavin	0.165900667	0.008889424	5.358281297
4	Particular compound	0.2079905	0.018056439	8.681376744
5	Gallic acid	0.153215833	0.009088662	5.931933746
6	Acetate	0.023965633	0.000705873	2.945356862
7	β-glucose	0.152879667	0.010514837	6.877852023
8	Fructose	0.306789667	0.030803153	10.04047945

S4 HPTLC and adulteration statistic

Table.4 List of SPE samples

Vial ID	Track Sample Name	Volume
C60	*R. crenulata* 60 % MeOH elution solvent	5.0 µL
S60	*R. sachalinensis* 60 % MeOH elution solvent	5.0 µL
R60	*R. rosea* 60 % MeOH elution solvent	5.0 µL

Table.5 List of raw material samples

Vial ID	Track Sample Name	Volume
RR1	*Rhodiola* crude root 1	5.0 µL
RR2	*Rhodiola* crude root 2	5.0 µL
RR3	*Rhodiola* crude root 3	5.0 µL
RR4	*Rhodiola* crude root 4	5.0 µL
RR5	*Rhodiola* crude root 5	5.0 µL
RR6	*Rhodiola* crude root 6	5.0 µL
RR7	*Rhodiola* crude root 7	5.0 µL
RR8	*Rhodiola* crude root 8	5.0 µL
RR9	*Rhodiola* crude root 9	5.0 µL

Vial ID	Track Sample Name	Volume
RR10	*Rhodiola* crude root 10	5.0 µL
RR11	*Rhodiola* crude root 11	5.0 µL
RR12	*Rhodiola* crude root 12	5.0 µL
RR13	*Rhodiola* crude root 13	5.0 µL
RR14	*Rhodiola* crude root 14	5.0 µL
RR15	*Rhodiola* crude root 15	5.0 µL
RR16	*Rhodiola* crude root 16	5.0 µL
RR17	*Rhodiola* crude root 17	5.0 µL
RR18	*Rhodiola* crude root 18	5.0 µL
RR19	*Rhodiola* crude root 19	5.0 µL

Vial ID	Track Sample Name	Volume
RR20	*Rhodiola* crude root 20	5.0 µL
RR21	*Rhodiola* crude root 21	5.0 µL
RR22	*Rhodiola* crude root 22	5.0 µL
RR23	*Rhodiola* crude root 23	5.0 µL
RR25	*Rhodiola* crude root 25	5.0 µL
RR26	*Rhodiola* crude root 26	5.0 µL
RR27	*Rhodiola* crude root 27	5.0 µL
RR30	*Rhodiola* crude root 30	5.0 µL
RR31	*Rhodiola* crude root 31	5.0 µL
RR32	*Rhodiola* crude root 32	5.0 µL

Vial ID	Track Sample Name	Volume
RR33	*Rhodiola* crude root 33	5.0 µL
RR34	*Rhodiola* crude root 34	5.0 µL
RR35	*Rhodiola* crude root 35	5.0 µL
RR36	*Rhodiola* crude root 36	5.0 µL
RR37	*Rhodiola* crude root 37	5.0 µL
RR38	*Rhodiola* crude root 38	5.0 µL
RR40	*Rhodiola* crude root 40	5.0 µL
RR46	*Rhodiola* crude root 46	5.0 µL
RR58	*Rhodiola* crude root 58	5.0 µL
RR61	*Rhodiola* crude root 61	5.0 µL

Vial ID	Track Sample Name	Volume
RR60	*Rhodiola* crude root 60	5.0 µL
RR59	*Rhodiola* crude root 59	5.0 µL
RR63	*Rhodiola* crude root 63	5.0 µL
RR62	*Rhodiola* crude root 62	5.0 µL
RR64	*Rhodiola* crude root 64	5.0 µL
RR65	*Rhodiola* crude root 65	5.0 µL

Table.6 Adulteration statistic of all *Rhodiola* samples. (through comparison with *R. crenulata* or *R. rosea*)

Serial. No	Sample. Ref	labelled as	Adulteration	Identified as
1	R1	*Rhodiola rosea*	Yes	not specified
2	R2	*Rhodiola rosea*	Yes	*Rhodiola crenulata*
3	R3	*Rhodiola sachalinensis*	No	–
4	R4	*Rhodiola crenulata*	Yes	*Rhodiola serrata*
5	R5	*Rhodiola rosea*	Yes	–
6	R6	*Rhodiola sachalinensis*	Yes	*Rhodiola serrata*
7	R7	*Rhodiola rosea*	Yes	*Rhodiola crenulata*
8	R8	*Rhodiola rosea*	Yes	*Rhodiola serrata*
9	R9	*Rhodiola sachalinensis*	No	–

Serial. No	Sample. Ref	labelled as	Adulteration	Identified as
10	R10	*Rhodiola quadrifida*	Yes	*Rhodiola serrata*
11	R11	*Rhodiola rosea*	Yes	*Rhodiola crenulata*
12	R12	*Rhodiola crenulata*	Yes	*Rhodiola serrata*
13	R13	*Rhodiola crenulata*	No	–
14	R14	*Rhodiola crenulata*	Yes	not specified
15	R15	*Rhodiola* (not specified)	–	not specified
16	R16	*Rhodiola* (not specified)	–	*Rhodiola serrata*
17	R17	*Rhodiola* (not specified)	–	*Rhodiola gelida*
18	R18	*Rhodiola* (not specified)	–	not specified
19	R19	*Rhodiola* (not specified)	–	*Rhodiola crenulata*

Serial. No	Sample. Ref	labelled as	Adulteration	Identified as
20	R20	*Rhodiola* (not specified)	–	not specified
21	R21	*Rhodiola* (not specified)	–	*Rhodiola crenulata*
22	R22	*Rhodiola* (not specified)	–	*Rhodiola crenulata*
23	R23	*Rhodiola* (not specified)	–	*Rhodiola crenulata*
24	R24	*Rhodiola rosea*	No	–
25	R25	*Rhodiola rosea*	No	–
26	R26	*Rhodiola* (not specified)	–	*Rhodiola crenulata*
27	R27	*Rhodiola* (not specified)	–	
28	R30	*Rhodiola crenulata*	–	–
29	R31	*Rhodiola sachalinensis*	–	–

Serial. No	Sample. Ref	labelled as	Adulteration	Identified as
30	R32	*Rhodiola quadrifida*	Yes	*Rhodiola serrata*
31	R33	*Rhodiola rosea*	Yes	*Rhodiola crenulata*
32	R34	*Rhodiola quadrifida*	Yes	*Rhodiola serrata*
33	R35	*Rhodiola crenulata*	No	–
34	R36	*Rhodiola* (not specified)	–	*Rhodiola crenulata*
35	R37	*Rhodiola rosea*	Yes	not specified
36	R38	*Rhodiola* (not specified)	–	*Rhodiola crenulata*
37	R40	*Rhodiola crenulata*	No	*Rhodiola crenulata*
38	R58	*Rhodiola rosea*	No	–
39	R59	*Rhodiola* (not specified)	–	*Rhodiola serrata*

Serial. No	Sample. Ref	labelled as	Adulteration	Identified as
40	R60	*Rhodiola* (not specified)	–	*Rhodiola crenulata*
41	R61	*Rhodiola* (not specified)	–	not specified
42	R62	*Rhodiola* (not specified)	–	*Rhodiola crenulata*
43	R63	*Rhodiola* (not specified)	–	*Rhodiola crenulata*
44	R64	*Rhodiola* (not specified)	–	not specified
45	R65	*Rhodiola* (not specified)	–	*Rhodiola crenulata*
